# A Case of Sudden-Onset Flaccid Paralysis In a Previously Healthy Person

**DOI:** 10.7759/cureus.37906

**Published:** 2023-04-20

**Authors:** Femina Patel, Chris Mehdizadeh, Prashila Amatya, Parash Bhakta, Estevan Torrez Andia

**Affiliations:** 1 Internal Medicine, University of California, Riverside School of Medicine, Riverside, USA; 2 Internal Medicine, University of California, Riverside College of Natural and Agricultural Sciences, Riverside, USA; 3 Internal Medicine, University of California, Riverside University Health System, Riverside, USA

**Keywords:** hypokalemic periodic paralysis, paraplegia in young, sudden onset weakness, hypokalemia, flaccid paralysis

## Abstract

Flaccid paralysis is a neurological syndrome characterized by weakness and paralysis of the limbs, followed by reduced muscle tone. Common causes of flaccid paralysis include blockage of the anterior spinal artery, trauma to the spinal cord, cancer, arterial disease, or thrombosis. A potential differential diagnosis in a 35-year-old male presenting with sudden-onset flaccid paralysis with no history of trauma is hypokalemic periodic paralysis. Treatment with potassium can alleviate symptoms in affected patients. .

## Introduction

Hypokalemic Periodic Paralysis (HPP) should be considered when making differential diagnoses for the presentation of sudden onset weakness or paralysis, especially among patients with no significant history and risk factors for stroke or trauma. HPP is a rare skeletal muscle disorder that causes abrupt painless muscle weakness or paralysis due to a defect in muscle channel ions. We present such a case in a 35-year-old male with hypertension and chronic back pain presented with sudden onset weakness in the upper and lower limbs likely due to HPP, exacerbated by corticosteroid injection and diuretics, managed with potassium replacement therapy resulting in complete resolution of symptoms.

## Case presentation

A 35-year-old male with a medical history of hypertension and chronic back pain presented to the emergency department with sudden onset weakness in the upper and lower limbs. He went to sleep the night prior with no weakness and awoke in the middle of the night, unable to move his bilateral upper and lower extremities. The patient denied any recent heavy exercise, fasting, high-carbohydrate meals, or any other lifestyle changes. He had no respiratory or swallowing difficulty and could move his neck and facial muscles. His sensation was grossly intact. He had received a steroid injection for back pain that same day. The patient reported a similar episode of sudden onset flaccid paralysis five years ago, resolved after intravenous potassium replenishment. He denied any similar episodes in the family. He denied recent fever, nausea, vomiting, diarrhea, or changes in urinary frequency. His home medications included hydrochlorothiazide and losartan.

**Figure 1:**
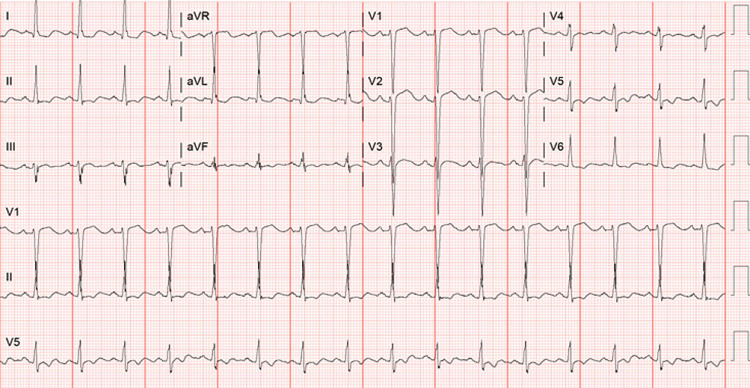
Initial electrocardiogram at presentation showing sinus rhytym EKG  showed sinus rhythm and ST/T wave changes in the lateral leads, which were not concerning for ischemia.

On arrival, he was hypertensive and tachycardic. His lab work was unremarkable except for a WBC of 24 x 10^9^/L, hemoglobin (Hb) of 18.1 g/L, potassium of 2.2 mmol/L (normal range: 3.5-5.0 mmol/L), blood glucose of 413 mg/dL, HbA1c of 5.3%, and lactic acid of 3.0 U/L. Other laboratory parameters were within normal limits, including liver function tests, thyroid function tests, and serum electrolytes. EKG showed sinus rhythm with no ischemic changes (Figure 1). The urine drug screen and urinalysis were unremarkable.

The patient was started on intravenous hydration, intravascular ceftriaxone 1 g after obtaining a blood culture, and intravenous (IV) potassium chloride (KCl) replacement in addition to oral KHCO_3_. In the next 24 hours, his potassium level reached within the normal limit, and supplementation was stopped. He received a total of 150 mEq of potassium during his hospital stay. He regained complete muscle strength and was able to ambulate without assistance. He was discharged with no neurological deficit. He was advised to increase his intake of potassium-rich foods and avoid triggers such as stress, alcohol, and strenuous exercise. Also, he was advised for an outpatient genetic workup.

## Discussion

HPP is a neuromuscular disorder related to a defect in potassium regulation due to sodium or calcium channel abnormalities. Prevalence is 1 in 100,000. The disease usually presents in the second or third decade of life and affects males more commonly than females. It may occur sporadically or in the form of Familial Hypokalemic Paralysis (FHP), which appears as the result of autosomal dominant inheritance. This is often caused by a missense mutation in a positively charged residue on either the L-type calcium channel or a sodium channel. In either case, a gating current prevents excitation of the skeletal muscle and causes flaccid paralysis because of sarcolemmal failure [1].

Precipitating factors for acute episodes of paralysis may include infection, stress, or medication (glucocorticoids, insulin, and diuretics). This patient's attack was likely due to the recent steroid injection in combination with hydrochlorothiazide. Steroids may cause potassium depletion by acting as agonists at mineralocorticoid receptors in the kidney collecting duct and causing increased activity of the H/K ATPase pump [2]. Thiazide diuretics such as hydrochlorothiazide may cause potassium depletion by increasing the volume of fluid and sodium delivered to the collecting duct, thus enhancing aldosterone's effect, which results in potassium wasting [3].

Diagnosis of hypokalemic periodic paralysis is based on clinical presentation, serum electrolyte levels, and exclusion of other neuromuscular disorders. Treatment involves prompt correction of hypokalemia with oral or intravenous potassium supplementation to avoid adverse cardiac, renal, or neurologic complications. When possible, address the underlying cause along with lifestyle modification to prevent the recurrence of attacks. Long-term complications may include cardiac arrhythmias due to hypokalemia and/or respiratory muscle paralysis and consequent respiratory insufficiency.

## Conclusions

Hypokalemic periodic paralysis is a rare but potentially debilitating disorder that can be diagnosed based on a careful clinical assessment and laboratory investigations. Prompt initiation of potassium supplementation is essential in managing acute attacks, and long-term management involves lifestyle modifications to prevent the recurrence of attacks. Although our patient's circumstances due to insurance did not allow for a definitive diagnosis via genetic testing, we strongly suspect hypokalemic periodic paralysis caused his episode.
